# Global analysis of DNA methylation in early-stage liver fibrosis

**DOI:** 10.1186/1755-8794-5-5

**Published:** 2012-01-27

**Authors:** Yoko Komatsu, Tsuyoshi Waku, Naoya Iwasaki, Wakana Ono, Chie Yamaguchi, Junn Yanagisawa

**Affiliations:** 1 Graduate School of Life and Environmental Sciences, University of Tsukuba, Tsukuba Science City, Ibaraki 305-8572, Japan; 2 Center for Tsukuba Advanced Research Alliance (TARA), Graduate School of Comprehensive Human Sciences, University of Tsukuba, Tsukuba Science City, Ibaraki 305-8572, Japan

## Abstract

**Background:**

Liver fibrosis is caused by chemicals or viral infection. The progression of liver fibrosis results in hepatocellular carcinogenesis in later stages. Recent studies have revealed the importance of DNA hypermethylation in the progression of liver fibrosis to hepatocellular carcinoma (HCC). However, the importance of DNA methylation in the early-stage liver fibrosis remains unclear.

**Methods:**

To address this issue, we used a pathological mouse model of early-stage liver fibrosis that was induced by treatment with carbon tetrachloride (CCl_4_) for 2 weeks and performed a genome-wide analysis of DNA methylation status. This global analysis of DNA methylation was performed using a combination of methyl-binding protein (MBP)-based high throughput sequencing (MBP-seq) and bioinformatic tools, IPA and Oncomine. To confirm functional aspect of MBP-seq data, we complementary used biochemical methods, such as bisulfite modification and *in-vitro*-methylation assays.

**Results:**

The genome-wide analysis revealed that DNA methylation status was reduced throughout the genome because of CCl_4 _treatment in the early-stage liver fibrosis. Bioinformatic and biochemical analyses revealed that a gene associated with fibrosis, *secreted phosphoprotein 1 *(*Spp1*), which induces inflammation, was hypomethylated and its expression was up-regulated. These results suggest that DNA hypomethylation of the genes responsible for fibrosis may precede the onset of liver fibrosis. Moreover, *Spp1 *is also known to enhance tumor development. Using the web-based database, we revealed that *Spp1 *expression is increased in HCC.

**Conclusions:**

Our study suggests that hypomethylation is crucial for the onset of and in the progression of liver fibrosis to HCC. The elucidation of this change in methylation status from the onset of fibrosis and subsequent progression to HCC may lead to a new clinical diagnosis.

## Background

Fibrosis is one of the most severe systemic diseases and is characterized by excessive accumulation of fibrous connective tissues, such as collagen, induced by acute or chronic injury [1]. Fibroproliferative diseases occur throughout the body, including in the lungs, kidneys, and liver. The progression of fibrosis leads to the failure of the physiological functions of tissues. Liver fibrosis, in particular, has been extensively investigated because its progression results in hepatocellular carcinoma (HCC), which is the fifth most common cancer worldwide [2,3].

Currently, liver fibrosis is known to be a part of the dynamic process of continuous extracellular matrix (ECM) remodeling in chronic liver injury [4]. In liver fibrosis, a liver injury activates the Kupffer cells--resident macrophages of the liver sinusoids--thereby inducing inflammation [5]. This inflammatory response triggers the activation of hepatic stellate cells (HSCs), which play a key role in fibrogenesis by transdifferentiating into myofibroblasts [1]. The proliferation of myofibroblasts and stimulation of ECM synthesis, ultimately results in liver fibrosis.

Recently, the progression of liver fibrosis has been reported to be associated with hypermethylation of DNA [6]. HSC activation is inhibited by 5'-Azacytidine (5'-Aza), a DNA methylation inhibitor, resulting in the transdifferentiation of HSCs to myofibroblasts [7]. Furthermore, in other tissues such as renal, several studies using heterozygous mice revealed that the DNA methyltransferase *Dnmt1 *and its inhibitor 5'-aza ameliorate renal fibrosis by inhibiting proliferation of myofibroblasts [8,9]. These results indicate the pivotal role of DNA hypermethylation in the progression of both liver and renal fibrosis. However, the importance of DNA methylation in early-stage liver fibrosis remains unclear.

Here, we performed a global analysis of DNA methylation during the onset of liver fibrosis. A mouse model treated with carbon tetrachloride (CCl_4_) was used as a model of liver fibrosis. Analysis of the CCl_4_-treated mouse livers revealed symptoms of early-stage liver fibrosis. To analyze the genome-wide DNA methylation profile of this CCl_4_-induced early-stage liver fibrosis, we used a combination of methyl-binding protein (MBP)-based precipitation (MBP-IP) and high-throughput DNA sequencing (MBP-seq). This genome-wide analysis can reveal hypo- and hypermethylated sites (125 and 88, respectively) in the genomic DNA. Analyzing the MBP-seq data, we revealed that the DNA methylation status was reduced throughout the genome, and that the enhancer of *secreted phosphoprotein 1 *(*Spp1*), also known as *osteopontin*, was hypomethylated. Two bioinformatics tools, IPA and Oncomine, indicated that *Spp1 *is related to liver fibrosis and inflammation. Using biochemical methods, such as bisulfite modification and *in-vitro*-methylation assays, we confirmed that the hypomethylation of *Spp1 *enhancer up-regulates its mRNA. These results clearly indicate that hypomethylation of the genome may precede the onset of liver fibrosis. Moreover, *Spp1 *enhances tumor development. This suggests that hypomethylation during the early-stage liver fibrosis may be important in the development of the primary liver cancer HCC, which is an end-stage liver disease.

## Methods

### Animal models

Five-week-old male C57BL/6 wild-type mice were purchased from CLEA Japan Inc. To induce liver fibrosis, 2 ml/kg CCl_4 _mixed with olive oil was intraperitoneally administered 3 times per week for 2 weeks. All animal husbandry and animal experiments were performed in accordance with the guidelines of the University of Tsukuba's Regulation of Animal Experiments Committee. Enzymatic activities of the serum proteins, alanine aminotranferease (Alt) and aspartate aminotransferase (Ast), were measured using the Fuji dri-chem 3000 analyzer (Fuji Film).

### Cell culture

Mouse liver cell line, Hepa1-6 cells were maintained in Dulbecco's modified Eagle's medium supplemented with 10% fetal bovine serum (FBS). Buffalo Rat liver cell line, BRL-3 A were maintained in Ham's F12 medium with 10% FBS. 3uM of the 5-Aza deoxy derivative, 5-dAza-C, were treated with BRL-3 A for 36hr, and then harvested for indicated experiments.

### Histopathological examination

After 2 weeks with or without CCl_4 _treatment, liver tissues were fixed with formalin. Formalin-paraffin livers were cut into 5 μm thick. Hematoxylin and Eosin (H&E) staining, Masson's trichrome (MT) staining and Sirius red staining were performed according to the manufacturer's protocol.

### Methyl-binding protein (MBP)-based high throughput sequencing (MBP-seq)

The genomic DNA from each of 4 mouse livers treated with 2 ml/kg CCl_4 _for 2 weeks were pooled. The genomic DNA from 3 mouse livers treated with olive oil for 2 weeks was pooled and used as control. The genomic DNA purified from the CCl_4_-treated mouse liver specimens was randomly fragmented into 50-350 bp lengths as described in the SOLiD 5500xl fragment library protocol. The fragmented DNA was then subjected to the MethylMiner methylated DNA enrichment kit according to the manufacturer's protocol. These methylated fragments were eluted with 1000 mM NaCl and used to construct standard fragment libraries using a combination of adaptor ligation and nick translation (SOLiD Fragment Library Construction Kit, Invitrogen). Each DNA library was selected on the basis of size (inserts were approximately 500 bp) by AgencourtAMPure XP (Beckman) before PCR amplification, bead attachment, and emulsion PCR. Libraries were sequenced on a SOLiD 5500xl Analyzer (Applied Biosystems). The resulting tag sequences and quality files were mapped onto the mouse genome (NCBI Build 37, UCSC mm9) using Lifescope version 2.0 (Life Technologies), and peaks were detected using the Genomics Workbench version 4.7.2 (CLC Bio). Parameters for peak mapping and detection are described for details below. Gene annotation of sequence peaks was performed through the BioMart website version 0.7 (http://www.biomart.org). The chromosomal distribution of the MBP-seq peaks was described using R version 1.4 (http://www.r-project.org). UCSC Genome Browser (http://genome.ucsc.edu) [10] was used to obtain data on the epigenetic markers and to display epigenetic features around the target genome locus.

### *In silico *functional analysis and network prediction by ingenuity pathway analysis (IPA)

The bioinformatics tool IPA version 1.0 (http://www.ingenuity.com) was used for *in silico *analysis of the MBP-seq data in the context of known functions and pathways using the Ingenuity Pathways Knowledge Base as a reference set, filtering for molecules and relationships associated with physiological and pathological processes of the liver. For the *in silico *functional analysis, a right-tailed Fisher's exact test was used to calculate the *p*-value determining the probability that the hepatotoxic function assigned to that data set was owing to chance alone.

### Oncomine data analysis

The web-based human cancer microarray database Oncomine (https://www.oncomine.com) was used to analyze the mRNA expression of target genes associated with HCC identified in three studies [11-13]. Details of standardized normalization techniques and statistical calculations can be found on the Oncomine website (https://www.oncomine.com) [14]. In brief, Student's *t*-test was performed to generate a *p*-value indicative of the significance of an observation. The lower the *p*-value, the more confidence in the difference between the groups. The Student's *t*-test statistic provided for the Oncomine visualizations reflects the magnitude of the difference between groups. Fold change is the magnitude of difference between the primary class and the other control classes, shown on a linear scale. An over-expression fold change is designated with a positive number.

### MBP-based precipitation and quantitative PCR (qPCR) of methylated DNA using MBD-IP

The purified genomic DNA from the mouse livers was randomly fragmented into 50-350 bp lengths as described in the SOLiD 5500xl fragment library protocol. The fragmented DNA was then subjected to the MethylMiner methylated DNA enrichment kit, according to the manufacturer's protocol (Invitrogen). qPCR was then performed to amplify and quantify fragments representative of the methylated genome using the Thermal Cycle Dice TP800 (TaKaRa) and SYBR Premix Ex Taq (TaKaRa). The primer sequences and genome locus used for MBP-IP are summarized in Additional file 1.

### Quantitative PCR (qPCR)

qPCR was performed as described previously [15]. Tissues were homogenized in 1 ml of Sepazol and total RNA was extracted according to the manufacturer's instructions (Nacalai Tesque). cDNA was synthesized from total RNA using RevatraAce reverse transcriptase (TOYOBO) and oligodT primer. qPCR was performed to amplify and quantify fragments representative of the indicated mRNA expression using a Thermal Cycle Dice TP800 (TaKaRa) and SYBR Premix Ex Taq (TaKaRa). *Cyclophilin *and *Gapdh *was used as the normalization control (Additional file 2). The primer sequences for qPCR are summarized in Additional file 1.

### *In-vitro*-methylation assay

The DNA fragments encoding *Spp1 *enhancer region (chr5:104846058-104846328) was amplified by PCR. The *Spp1 *enhancer fragment was methylated by the DNA methylase Sss1 (2U enzyme/10ug DNA), and then cloned into a pGL3-basic reporter plasmid. Each plasmid harboring methylated or unmethylated fragment was directly transfected into Hepa1-6 cells by Lipofectamine 2000 (Invitrogen), according to the manufacturer's instruction. Twenty-four hours after transfection, luciferase assays were performed by Dual-Luciferase Reporter 1000 Assay System (Promega), according to the manufacturer's instruction. phRG-TK (Promega) was used as a reference plasmid to normalize transfection efficiency.

### Bisulfite modification assay

The purified genomic DNA from the mouse livers were denatured in 0.3M NaOH for 20min at 37°C. Then 3.6N sodium bisulfite and 10mM hydroquinone solution were added. Samples were incubated in 1min at 95°C then 12hr at 50°C. Salts were removed using the Wizard DNA Clean-Up System (Promega) and desulfonated in 0.3M NaOH at room temperature for 5 min. Then, the Spp1 enhancer region was amplified by PCR and cloned into pGEM-T Easy Vector System (Promega). The inserts were sequenced to identify the methylated and unmethylated sites. The primer sequences used for amplification are summarized in Additional file 1.

### Data access

Sequence data of CCl_4_-treated and control samples from this study has been deposited to the NCBI Sequence Read Archive (http://www.ncbi.nlm.nih.gov/sra) under accession no. SRA048978 and SRA048984, respectively.

## Results

### Mouse model of CCl_4_-induced early-stage liver fibrosis

**Figure 1:**
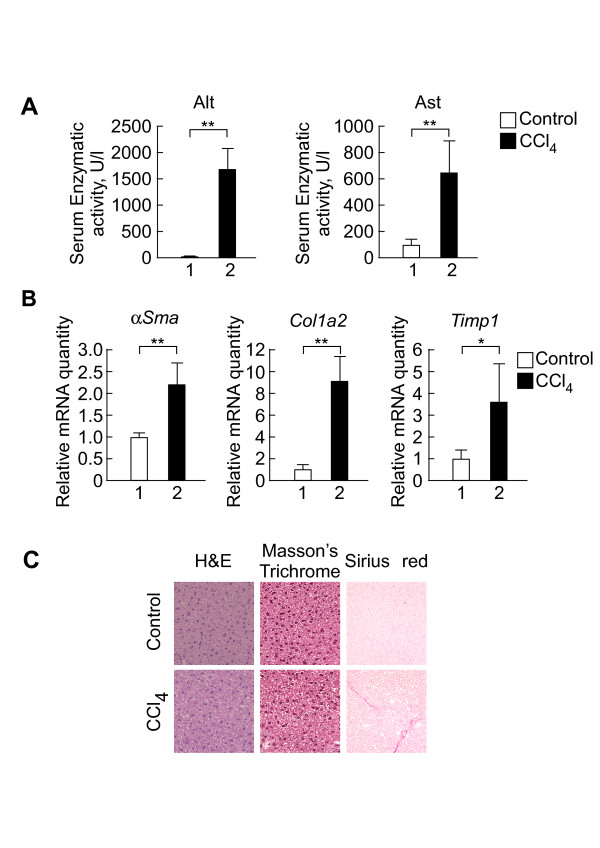
**Validation of a mouse model of CCl**_**4**_**-induced early-stage liver fibrosis**. **(A) **The effect of CCl_4 _treatment on enzymatic activities of serum Alt and Ast. The vertical axis represents each enzymatic activity (U/I) plus the standard deviation (**p-value < 0.01, n = 3-4). **(B) **The effect of CCl_4 _treatment on mRNA levels of collagen-related genes. mRNA levels of ECM-related genes, such as *αSma, Col1a2*, and *Timp1*, were determined by qPCR. *Cyclophilin *was used as the normalization control. The vertical axes represent the relative mRNA quantity plus the standard deviation (**p-value < 0.01, n = 3-4). **(C) **Histological changes induced by CCl_4 _in mouse livers. Paraffin-fixed liver section stained with H&E, MT, and Sirius-red (original magnification ×400).

To develop early-stage liver fibrosis, mice were administered 2 ml/kg CCl_4 _for 2 weeks (see Methods for details). To determine whether this treatment induced liver fibrosis, we used blood samples to investigate two enzymatic activities of well-known fibrosis-indicative serum parameters--Alt and Ast. We found that CCl_4 _treatment increased enzymatic activities of both Alt and Ast (Figure 1A). Furthermore, we found that well-known fibrotic markers, such as α*Sma, Col1a2*, and *Timp1*, were up-regulated in the CCl_4_-treated livers (Figure 1B). To confirm histopathological change in the livers following CCl_4 _treatment, we used MT and Sirius-red staining, which are commonly used to detect collagen accumulation in liver tissue. Although MT staining did not reveal histological changes, Sirius-red staining revealed an increased distribution of collagen (Figure 1C). Given that MT staining is known to detect continuous ECM whereas Sirius-red staining specifically detects collagen [16], these results suggest that 2 weeks of CCl_4 _treatment induces fiber production, but not fibroblast growth. This suggests that treatment with CCl_4 _induced the development of early-stage liver fibrosis.

### Genome-wide DNA methylation profile of the CCl_4_-treated livers

To investigate the effect of CCl_4 _treatment on DNA methylation status in early-stage liver fibrosis, we attempted to profile the genome-wide DNA methylation of the CCl_4_-treated liver tissues. The genomic DNA was fragmented, and the methylated DNA fragment was precipitated by MBP. The methylated DNA fraction was then subjected to high-throughput sequencing using a SOLiD 5500xl. These sequence reads were then mapped onto the mouse genome (NCBI37/mm9) using the MethylMiner methylated DNA enrichment kit and Lifescope. We obtained 7,612,236 reads from the CCl_4 _sample, and 24,584,122 reads from the control [17]. To find the significant peak, these data were further analyzed using the ChIP-seq tool in Genomics Workbench with the mouse genome annotated using the BioMart website. The peak-finding algorithm included the following four steps: 1) Calculate the null distribution of the background sequencing signal; 2) Scan the mappings to identify candidate peaks with a higher read count than expected from the null distribution; 3) Merge overlapping candidate peaks; 4) Refine the set of candidate peaks based on the count and the spatial distribution of forward and backward reads within the peaks. The estimate for the null distribution of coverage and the calculation of the false discovery rate (FDR) were based on the window size and maximum FDR (%) parameters. The window size specifies the width of the window that is used to count reads during estimation of the null distribution as well as during subsequent scanning for candidate peaks. Maximum FDR indicates the maximum proportion of false positive peaks that are acceptable among the called peaks. In this study, when only MBP samples were used, each negative binomial distribution was fitted to the counts from the low coverage regions. This distribution was used as the null distribution to obtain the number of windows with a particular count of reads expected in the absence of significant binding. By comparing the number of windows with the specific count we expected to observe under the null distribution and the number we actually observed in our data, we can calculate FDR for a given read count and window size as the "fraction of windows with a read count expected under the null distribution/fraction of windows with the observed read count". In this study, we set window size and FDR to 300 bp and 1%, respectively. We identified 125 and 88 peaks in the CCl_4_-treated and control samples, and summarized all peaks with CpG annotation and several statistic values, including FDR, normalized difference, and Wilcoxon filter *p*-value, in Additional file 3.

### Chromosomal distribution and genomic features of the MBP-seq peaks

**Figure 2:**
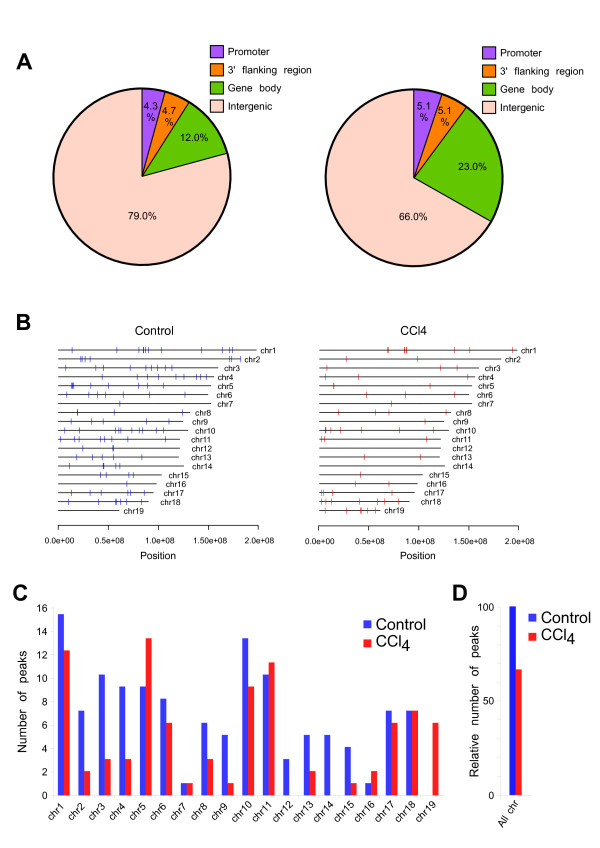
**DNA methylation profile of the liver genome**. **(A) **Pie chart representing the proportions of genomic features with CCl_4_- and control-specific peaks. Gene body was defined as transcribed region, involving exon and intron. Promoter was as 20 kbp regions upstream from transcriptional start sites (TSS). 3'-flanking region was as 20 kbp regions downstream from transcription termination sites (TTS). These definitions were determined by using the UCSC Genome browser database. Intergenic region represented other region these defined regions. **(B) **Genome-wide distribution of methylated sites. Red and blue bars represent methylated sites of the CCl_4_- and control-specific peaks, respectively. **(C) **Number of methylated sites in each chromosome. Red and blue bars represent the CCl_4_- and control-specific peaks, respectively. **(D) **Relative number of methylated sites in all chromosomes. CCl_4_-specific peaks in all chromosomes (red) were relatively compared with control ones (blue).

The pie chart in Figure 2A indicates the methylated sites in each genomic element, such as the transcription regulatory region including the promoter and the 3' flanking region, the gene body including the exon and intron, and the intergenic region (See Figure 2A legend for detail definition of these terms). Methylated sites in the gene body, the transcription regulatory region, and the intergenic region, were assigned (23.0/12.0%, 10.2/9.0%, and 66.0/79.0% in CCl_4_-treated/control samples, respectively) (Figure 2A). We found that almost methylated sites are specific to each sample (Additional file 3). Therefore, we considered CCl_4_-specific and control-specific methylated sites as CCl_4_-induced "hyper" and "hypo"methylation sites, respectively. The chromosomal distribution of the MBP peaks revealed that the number of control-specific methylation peaks was greater than that of CCl_4_-specific peaks in most chromosomes (Figure 2B-D). These results indicate that the onset of CCl_4_-induced early-stage liver fibrosis might be associated with global hypomethylation.

### *In silico *functional analysis of genes annotated by MBD-seq

**Figure 3:**
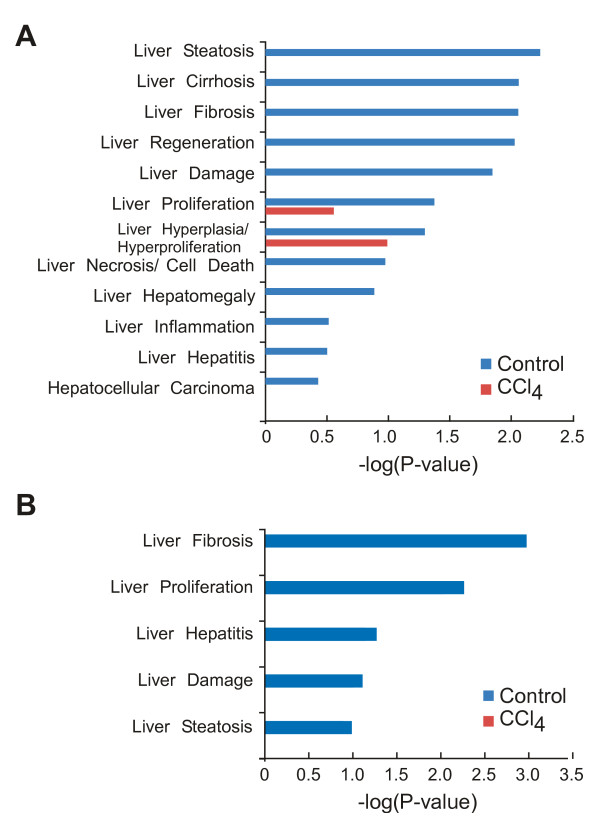
***In silico *functional analysis of methylated sites**. IPA analysis of all methylation peaks **(A)**, and annotated peaks among the promoters **(B)**. The horizontal axis represents the significance scores for each function as -log (*p*-value). Red and blue bars represent CCl_4 _and control samples, respectively.

Then, the functions of the genes with methylated sites, which are assigned to the gene body and the transcription regulatory region, are classified using *in silico *analysis software, IPA (See for details Methods for IPA analysis, and Discussion for the functional aspect of methylated sites within intergenic region). We found that cirrhosis, fibrosis, and HCC were identified only in the control sample (Figure 3A). Previous studies have indicated that the progression of liver fibrosis leads to cirrhosis and eventually to HCC, a primary hepatic cancer [18]. Furthermore, it has been known that aberrant regulation of gene expression is at the basis of many diseases, such as cancer [19]. These results suggest that aberrant DNA methylation causes dysfunction of gene transcription and may affect the onset of fibrosis and its progression to post-fibrotic diseases.

Using IPA, we then re-analyzed the annotated genes in the promoters, which is important for gene transcription. Intriguingly, liver fibrosis was detected as the predominant hepatotoxic function in the control sample (*p*-value = 1.06 × 10^-3^-6.37 × 10^-3^) (Figure 3B). Moreover, none of the liver diseases detected in control samples were detected in the CCl_4_-treated samples. IPA re-analysis revealed that this fibrotic function in the control sample was associated with one gene--*Spp1*.

In IPA, the *p*-value is calculated using a right-tailed Fisher's exact test to assess the probability that the association between the focal molecules in the experiment and a given function is owing to random chance. Smaller the *p*-value, lesser is the probability of random association. In general, *p*-values < 0.05 indicate a statistically significant, nonrandom association. Therefore, these results suggest that CCl_4_-induced hypomethylation of a regulatory region, such as the promoter or enhancer of *Spp1*, may result in the onset of liver fibrosis and its progression to post-fibrotic diseases such as cirrhosis and HCC through an increase in *Spp1 *expression.

### Epigenetic features and functional validations of the hypomethylated region upstream of *Spp1*

**Figure 4:**
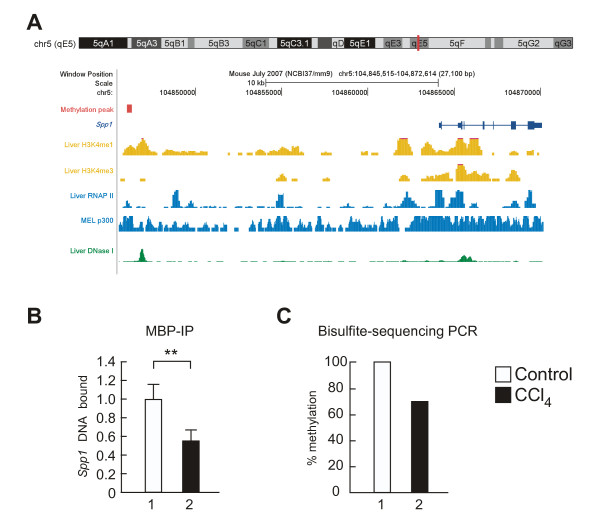
**Validation of the methylation site located upstream of the *Spp1 *TSS**. **(A) **Epigenetic features of the methylation site located upstream of the *Spp1 *TSS assessed using the UCSC Genome browser. The *Spp1 *gene body is shown in dark blue. The methylation site upstream of the *Spp1 *TSS is shown as a red rectangle on the chromosome diagram and on the custom track. ChIP-seq signals for H3K4 me1 and H3K4 me4 in the liver (orange), for RNAP II in the liver and p300 in MEL leukemia (light blue), and for the DNase I hotspot in the liver (green) are represented as density plots. **(B) **MBP-IP assay of the methylation status of the site located upstream of the *Spp1 *TSS. The CCl_4 _sample was normalized to the control sample. The vertical axis represents fold changes plus the standard deviation (**p-value < 0.01, n = 3-4). **(C) **Bisulfite modification assay of the methylation status of the site located upstream of the *Spp1 *TSS. The CCl_4 _sample was normalized to the control sample. The vertical axis represents percentage of methylated sites in the site located upstream of the *Spp1 *TSS (n = 10).

The hypomethylation peak was located approximately 18 kbp upstream of the transcription start site (TSS) of *Spp1 *(Figure 4A, **red**). We placed this MBP-seq peak into the UCSC genome browser and found that the hypomethylation site upstream of *Spp1 *has several chromatin features identified from studies by other groups, including: 1) mono- and trimethylation of histone 3 lysine 4 in the liver (H3K4 me1 and H3K4 me3) (Figure 4A, **orange**) [20]; 2) the binding of RNA polymerase II and related acetyltransferace p300 in the liver and in the MEL leukemia cell line (Figure 4A, **light blue**) [21]; and 3) it is a DNase I hotspot in the liver (Figure 4A, **green**) [22]. Previous studies have demonstrated that these epigenetic features are observed in the enhancer region [20,23-25], and that hypomethylation of an enhancer predominantly induces mRNA expression [26-28]. Thus, these epigenetic annotations lead to the hypothesis that this hypomethylated site may function as an enhancer that regulates *Spp1 *expression.

**Figure 5:**
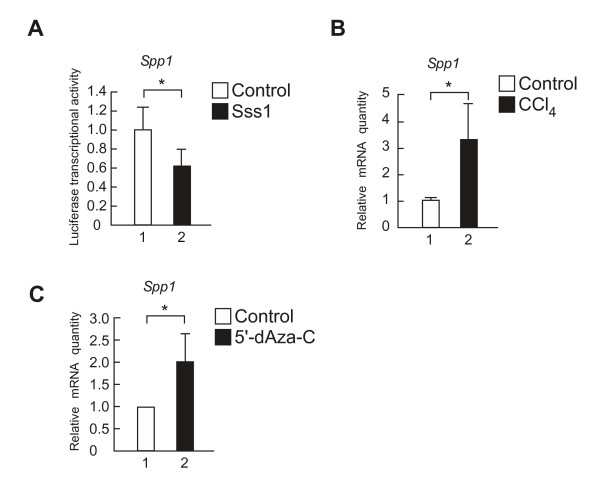
**Effect of the methylation site located upstream of the *Spp1 *TSS on *Spp1 *expression**. **(A) ***In-vitro*-methylation assay of the site located upstream of the *Spp1 *TSS. The fragment DNA of the site located upstream of the *Spp1 *TSS (chr5:104846058-104846328) was enzymatically methylated *in vitro *by Sss1 before ligation into pGL3-Basic. The vertical axis represents the luciferase activity plus the standard deviation. (*p-value < 0.05, n = 3). **(B) **qPCR assay of *Spp1 *mRNA expression. *Cyclophilin *was used as the normalization control. The vertical axis represents the relative mRNA quantity plus the standard deviation (*p-value < 0.05, n = 3-4). **(C) **The effect of 5-dAza-C treatment on expression of *Spp1 *mRNA. mRNA levels of *Spp1 *were determined by qPCR. *Gapdh *was used as the normalization control. The vertical axis represents the relative mRNA quantity plus the standard deviation (*p-value < 0.05, n = 3).

To confirm this hypothesis, we performed biochemical assays. Considering with the technical limitation of MBD-IP assay, a bisulfite modification assay was carried out to verify at a single-locus-based resolution (See Methods for details). Complementary using these assays, we confirmed that the site annotated by the sequence database was actually hypomethylated by CCl_4 _treatment (Figure 4B and 4C). We then examined whether the methylation on the upstream of *Spp1 *TSS will affect transcriptional activity. Luciferase assay were performed using *in vitro *methylated upstream of *Spp1 *TSS. This *in-vitro*-methylation assay revealed that methylation on upstream of *Spp1 *TSS down-regulated its mRNA levels (Figure 5A). We then performed a qPCR assay and revealed that *Spp1 *expression was increased by treatment with CCl_4 _(Figure 5B) and 5-dAza-C (Figure 5C), both of which induce hypomethylation upstream of *Spp1 *TSS. These results allow us to conclude that the upstream of *Spp1 *TSS functions as enhancer of *Spp1*. These results suggest that CCl_4 _treatment might up-regulate *Spp1 *expression in early-stage liver fibrosis through hypomethylation of the *Spp1 *enhancer.

### Functional significance of *Spp1 *in HCC

**Figure 6:**
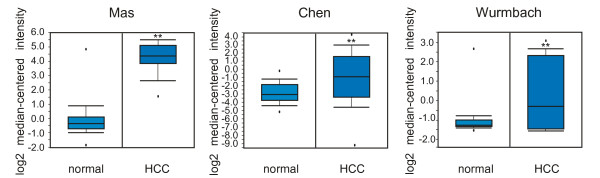
**Oncomine analysis of *Spp1 *mRNA expression in human HCC**. Box-plot diagrams were analyzed to compare the *Spp1 *mRNA levels in normal liver tissue with that in HCC using the Oncomine dataset from studies reported by Mas et al. (**p-value < 0.01), Chen et al. (**p-value < 0.01), and Wurmbach et al. (**p-value < 0.01). The vertical axis represents the log_2 _median value. The upper (75%) and lower (25%) quartiles are represented by the upper and lower borders of the boxes, respectively.

*In silico *analysis and functional validation of hypomethylation suggest that *Spp1 *expression might cause fibrosis-induced liver diseases, such as those shown in Figures 3A and 3B; this hypothesis is in agreement with previous studies [29]. Thus, to obtain functional insight into *Spp1 *in the end-stage liver disease HCC, we analyzed the mRNA level using the human cancer microarray database Oncomine. Analysis of studies by Mas et al. [11] , Chen et al. [12] , and Wurmbach et al. [13] revealed that *Spp1 *expression was higher in HCC than in normal liver tissues (Figure 6), suggesting that *Spp1 *might play a crucial role in HCC development as well as in the onset of fibrosis.

## Discussion

The DNA methylation status of well-known genes associated with fibrosis progression, such as Rasal1, Fli1, and Thy1, has been reported to increase along with fibrosis progression, which induces proliferation of fibroblasts and the production of collagen [9,30,31]. Furthermore, 5'-Aza, an inhibitor of DNA methylation, reportedly attenuates the progression of renal and liver fibrosis *in vivo *and *in vitro *[7,8]. These reports indicate the importance of hypermethylation of the genome in the progression of liver fibrosis.

In contrast to these previous reports, our analysis revealed that DNA methylation status was significantly reduced in CCl_4_-induced early-stage liver fibrosis. Detailed analyses revealed that in early-stage liver fibrosis, the *Spp1 *enhancer was hypomethylated and *Spp1 *expression was up-regulated. Previous findings from genetically engineered *Spp1-*knockout mice have shown that *Spp1 *plays an important role in both progression and reduction of liver injury and fibrosis [32,33]. Although the precise mechanism underlying the role of *Spp1 *in fibrosis remains unknown, *Spp1 *is a pivotal cytokine/chemokine generated by the Kupffer cells in response to liver damage [34] that induces inflammation [35], which is a contributing factor in liver fibrosis. Our results indicate that an epigenetic alteration, DNA hypomethylation of the *Spp1 *enhancer, may precede the up-regulation of *Spp1 *expression and induce the onset of CCl_4_-induced early-stage liver fibrosis. *Spp1 *is also a known enhancer of tumor development and metastasis [36]. Using the Oncomine microarray database, we demonstrated that *Spp1 *expression level is increased in HCC. It has been documented in primary gastric cancers, that major phenotypic change in cancer-associated myofibroblasts is a global reduction in DNA methylation [17]. This may also be indicated in liver, the importance of hypomethylation of the *Spp1 *enhancer in the progression of HCC.

Unlike *Spp1*, we found that almost DNA hypomethylation induced in early fibrosis was assigned in intergenic regions (Figure 2A). Recently, it has been reported that DNA hypomethylation causes genomic instability and alteration of gene transcription in several human cancers (i.e. colorectal and prostatic adenocarcinoma, breast cancer, intestinal type-gastric carcinoma, and HCC) [37,38]. These results indicate that DNA hypomethylation in intergenic regions may trigger the progression of cancer through genomic instability of cancer-related genes in addition to transcriptional regulation of those genes in the onset of liver fibrosis. On the other hand, 5'-Aza treatment successfully reduces cancer in mammals, involving human, suggesting that carcinogenesis results from DNA hypermethylation as well as DNA hypomethylation [38-40]. Therefore, how these distinct DNA modifications commonly progress cancer is most important study for epigenetic effects on diseases in the future.

## Conclusion

Although hypermethylation occurs during the progression of liver fibrosis, our results indicate the importance of hypomethylation in the onset of liver fibrosis. According to our results as well as other reports, it appears that DNA methylation status may change from hypo- to hypermethylation during the progression of liver fibrosis. Thus, hypomethylation in early-stage liver fibrosis may contribute to the onset and/or development of HCC.

## Abbreviations

α-Sma: α-Smooth muscle actin; Alt: Alanine aminotransferase; Ast: Aspartate aminotransferase; CCl_4_: Carbon tetrachloride; Col1a2: Collagen, type I, alpha 2; ECM: Extracellular matrix; Fli1: Friend leukemia integration 1; HCC: Hepatocellular carcinoma; Rasal1: RAS protein activator like 1; Spp1: Secreted phosphoprotein 1; Thy1: Thymocyte differentiation antigen 1; Timp1: Tissue inhibitor of metalloproteinase 1

## Competing interests

The authors declare that they have no competing interests.

## Authors' contributions

YK and NI performed the animal experiments and biochemical analyses. TW performed the high-throughput sequencing and bioinformatic analysis. WO and CY supported these analyses. All authors wrote, read, and approved the final manuscript.

## Pre-publication history

The pre-publication history for this paper can be accessed here:

http://www.biomedcentral.com/1755-8794/5/5/prepub

## Supplementary Material

Additional file 1 **Table of primer sets used in this study**. These qPCR primer sets were used in Figures 1B, 5B and C. Click here for file

Additional file 2 **mRNA levels of reference genes**. *Cyclophilin *were used to normalize genes in Figure 1B and 5B. *Gapdh *were used to normalize genes in Figure 5C. Click here for file

Additional file 3 **Statistics and annotation of MBP-seq peaks**. This table was generated by Genomics Workbench, and used in Figures 2, 3, and 4A. Click here for file

## References

[B1] Bataller R Brenner DA Liver fibrosis The Journal of clinical investigation 2005 115 2 209 218 15690074 10.1172/JCI24282 PMC546435

[B2] Iredale JP Models of liver fibrosis: exploring the dynamic nature of inflammation and repair in a solid organ The Journal of clinical investigation 2007 117 3 539 548 10.1172/JCI30542 17332881 PMC1804370

[B3] Llovet JM Updated treatment approach to hepatocellular carcinoma Journal of gastroenterology 2005 40 3 225 235 10.1007/s00535-005-1566-3 15830281

[B4] Friedman SL Liver fibrosis -- from bench to bedside Journal of hepatology 2003 38 Suppl 1 S38 53 12591185 10.1016/s0168-8278(02)00429-4

[B5] Friedman SL Mechanisms of disease: Mechanisms of hepatic fibrosis and therapeutic implications Nature clinical practice 2004 1 2 98 105 10.1038/ncpgasthep0055 16265071

[B6] Friedman SL Mechanism of Hepatic Fibrogenesis Gastroenterology 2008 134 1655 1669 10.1053/j.gastro.2008.03.003 18471545 PMC2888539

[B7] Mann J Oakley F Akiboye F Elsharkawy A Thorne AW Mann DA Regulation of myofibroblast transdifferentiation by DNA methylation and MeCP2: implications for wound healing and fibrogenesis Cell death and differentiation 2007 14 2 275 285 10.1038/sj.cdd.4401979 16763620

[B8] Bechtel W McGoohan S Zeisberg EM Muller GA Kalbacher H Salant DJ Muller CA Kalluri R Zeisberg M Methylation determines fibroblast activation and fibrogenesis in the kidney Nature medicine 2010 16 5 544 550 10.1038/nm.2135 20418885 PMC3106179

[B9] Ortiz A Ucero AC Egido J Unravelling fibrosis: two newcomers and an old foe Nephrol Dial Transplant 2010 25 11 3492 3495 10.1093/ndt/gfq518 20833689

[B10] Kent WJ Sugnet CW Furey TS Roskin KM Pringle TH Zahler AM Haussler D The human genome browser at UCSC Genome research 2002 12 6 996 1006 12045153 10.1101/gr.229102 PMC186604

[B11] Mas VR Maluf DG Archer KJ Yanek K Kong X Kulik L Freise CE Olthoff KM Ghobrial RM McIver P Genes involved in viral carcinogenesis and tumor initiation in hepatitis C virus-induced hepatocellular carcinoma Molecular medicine (Cambridge, Mass 2009 15 3-4 85 94 10.2119/molmed.2008.00110 PMC2605622 19098997

[B12] Chen X Cheung ST So S Fan ST Barry C Higgins J Lai KM Ji J Dudoit S Ng IO Gene expression patterns in human liver cancers Molecular biology of the cell 2002 13 6 1929 1939 10.1091/mbc.02-02-0023. 12058060 PMC117615

[B13] Wurmbach E Chen YB Khitrov G Zhang W Roayaie S Schwartz M Fiel I Thung S Mazzaferro V Bruix J Genome-wide molecular profiles of HCV-induced dysplasia and hepatocellular carcinoma Hepatology (Baltimore, Md 2007 45 4 938 947 10.1002/hep.21622 17393520

[B14] Rhodes DR Yu J Shanker K Deshpande N Varambally R Ghosh D Barrette T Pandey A Chinnaiyan AM ONCOMINE: a cancer microarray database and integrated data-mining platform Neoplasia (New York, NY 2004 6 1 1 6 10.1016/s1476-5586(04)80047-2 PMC1635162 15068665

[B15] Murayama A Ohmori K Fujimura A Minami H Yasuzawa-Tanaka K Kuroda T Oie S Daitoku H Okuwaki M Nagata K Epigenetic control of rDNA loci in response to intracellular energy status Cell 2008 133 4 627 639 10.1016/j.cell.2008.03.030 18485871

[B16] Fabre Y Bueno MR Rincon-Sanchez AR Saldana-Cortes J Vargas R Armendariz-Borumda J Mexican infants with extrahepatic biliary atresia display different fibrosis activity Hepatology Research 2004 28 79 86 10.1016/j.hepres.2003.10.004

[B17] Jiang L Gonda TA Gamble MV Salas M Seshan V Tu S Twaddell WS Hegyi P Lazar G Steele I Global hypomethylation of genomic DNA in cancer-associated myofibroblasts Cancer research 2008 68 23 9900 9908 10.1158/0008-5472.CAN-08-1319 19047171 PMC2670548

[B18] Bissell DM Hepatic fibrosis as wound repair: a progress report Journal of gastroenterology 1998 33 2 295 302 10.1007/s005350050087 9605966

[B19] Gallinari Paola Stefania Di Marco Jones Phillip Pallaro Michele Christian Steinkühler HDACs, histone deacetylation and gene transcription: from molecular biology to cancer therapeutics Cell Research 2007 17 195 211 17325692 10.1038/sj.cr.7310149

[B20] Heintzman ND Hon GC Hawkins RD Kheradpour P Stark A Harp LF Ye Z Lee LK Stuart RK Ching CW Histone modifications at human enhancers reflect global cell-type-specific gene expression Nature 2009 459 7243 108 112 10.1038/nature07829 19295514 PMC2910248

[B21] Euskirchen GM Rozowsky JS Wei CL Lee WH Zhang ZD Hartman S Emanuelsson O Stolc V Weissman S Gerstein MB Mapping of transcription factor binding regions in mammalian cells by ChIP: comparison of array- and sequencing-based technologies Genome research 2007 17 6 898 909 10.1101/gr.5583007 17568005 PMC1891348

[B22] Sabo PJ Kuehn MS Thurman R Johnson BE Johnson EM Cao H Yu M Rosenzweig E Goldy J Haydock A Genome-scale mapping of DNase I sensitivity in vivo using tiling DNA microarrays Nature methods 2006 3 7 511 518 10.1038/nmeth890 16791208

[B23] Bulger M Groudine M Enhancers: the abundance and function of regulatory sequences beyond promoters Developmental biology 2010 339 2 250 257 10.1016/j.ydbio.2009.11.035 20025863 PMC3060611

[B24] Heintzman ND Stuart RK Hon G Fu Y Ching CW Hawkins RD Barrera LO Van Calcar S Qu C Ching KA Distinct and predictive chromatin signatures of transcriptional promoters and enhancers in the human genome Nature genetics 2007 39 3 311 318 10.1038/ng1966 17277777

[B25] Pourcel C Tiollais P Farza H Transcription of the S Gene in Transgenic Mice Is Associated with Hypomethylation at Specific Sites and with Dnase I Sensitivity Journal of Virology 1990 64 2 931 935 2296089 10.1128/jvi.64.2.931-935.1990 PMC249193

[B26] Kelley DE Pollok BA Atchison ML Perry RP The coupling between enhancer activity and hypomethylation of kappa immunoglobulin genes is developmentally regulated Molecular and cellular biology 1988 8 2 930 937 3127693 10.1128/mcb.8.2.930-937.1988 PMC363225

[B27] Serandour AA Avner S Percevault F Demay F Bizot M Lucchetti-Miganeh C Barloy-Hubler F Brown M Lupien M Metivier R Epigenetic switch involved in activation of pioneer factor FOXA1-dependent enhancers Genome research 2011 21 4 555 565 10.1101/gr.111534.110 21233399 PMC3065703

[B28] Tokizane T Shiina H Igawa M Enokida H Urakami S Kawakami T Ogishima T Okino ST Li LC Tanaka Y Cytochrome P450 1B1 is overexpressed and regulated by hypomethylation in prostate cancer Clin Cancer Res 2005 11 16 5793 5801 10.1158/1078-0432.CCR-04-2545 16115918

[B29] Ramaiah SK Rittling S Pathophysiological role of osteopontin in hepatic inflammation, toxicity, and cancer Toxicol Sci 2008 103 1 4 13 17890765 10.1093/toxsci/kfm246

[B30] Sanders YY Pardo A Selman M Nuovo GJ Tollefsbol TO Siegal GP Hagood JS Thy-1 promoter hypermethylation: a novel epigenetic pathogenic mechanism in pulmonary fibrosis American journal of respiratory cell and molecular biology 2008 39 5 610 618 10.1165/rcmb.2007-0322OC 18556592 PMC2574530

[B31] Wang Y Fan PS Kahaleh B Association between enhanced type I collagen expression and epigenetic repression of the FLI1 gene in scleroderma fibroblasts Arthritis and rheumatism 2006 54 7 2271 2279 10.1002/art.21948 16802366

[B32] Lorena D Darby IA Gadeau AP Leen LL Rittling S Porto LC Rosenbaum J Desmouliere A Osteopontin expression in normal and fibrotic liver. altered liver healing in osteopontin-deficient mice Journal of hepatology 2006 44 2 383 390 10.1016/j.jhep.2005.07.024 16221502

[B33] Sahai A Malladi P Melin-Aldana H Green RM Whitington PF Upregulation of osteopontin expression is involved in the development of nonalcoholic steatohepatitis in a dietary murine model American journal of physiology 2004 287 1 G264 273 15044174 10.1152/ajpgi.00002.2004

[B34] Kawashima R Mochida S Matsui A YouLuTu ZY Ishikawa K Toshima K Yamanobe F Inao M Ikeda H Ohno A Expression of osteopontin in Kupffer cells and hepatic macrophages and Stellate cells in rat liver after carbon tetrachloride intoxication: a possible factor for macrophage migration into hepatic necrotic areas Biochemical and biophysical research communications 1999 256 3 527 531 10.1006/bbrc.1999.0372 10080931

[B35] Ramaiah SK Rittling S Role of osteopontin in regulating hepatic inflammatory responses and toxic liver injury Expert opinion on drug metabolism & toxicology 2007 3 4 519 526 10.1517/17425255.3.4.519 17696803

[B36] Ariztia EV Subbarao V Solt DB Rademaker AW Iyer AP Oltvai ZN Osteopontin contributes to hepatocyte growth factor-induced tumor growth and metastasis formation Experimental cell research 2003 288 2 257 267 10.1016/S0014-4827(03)00118-6 12915117

[B37] Eden Amir Gaudet François Waghmare Alpana Jaenisch Rudlf Chromosomal Instability and Tumors Promoted by DNA Hypomethylation Science 2003 300 18 10.1126/science.1083557 12702868

[B38] Tischoff Iris Tannpfel Andrea DNA methylation in hepatocellular carcinoma World Journal of Gastroenterology 2008 14 11 1741 1748 10.3748/wjg.14.1741 18350605 PMC2695914

[B39] Gaudet François J Graeme Hodgson Eden Amir Laurie Jackson-Grusby Dausman Jessica Joe Gray W Leonhardt Heinrich Jaenisch Rudolf Induction of Tumors in Mice by Genomic Hypomethylation Science 2003 300 18 10.1126/science.1083558 12702876

[B40] Adam Karpf R David Jones A Reactivating the expression of methylation silenced genes in human cancer Oncogene 2002 21 5496 5503 10.1038/sj.onc.1205602 12154410

