# Supplementary figures and images for: Global analysis of DNA methylation in early-stage liver fibrosis
Source: BMC Med Genomics. 2012 Jan 27;5:5. doi: 10.1186/1755-8794-5-5 (PMC3295686; doi:10.1186/1755-8794-5-5)

## Additional file 2 - mRNA levels of reference genes

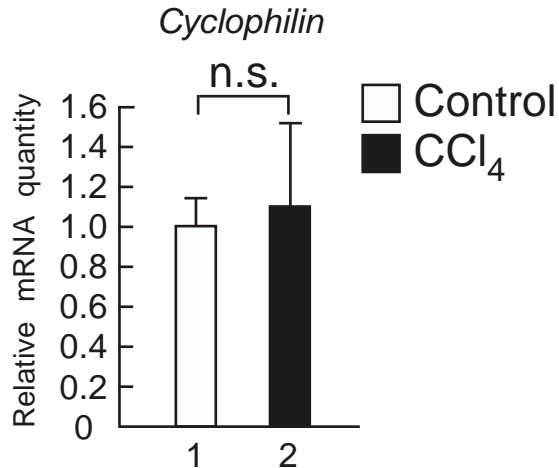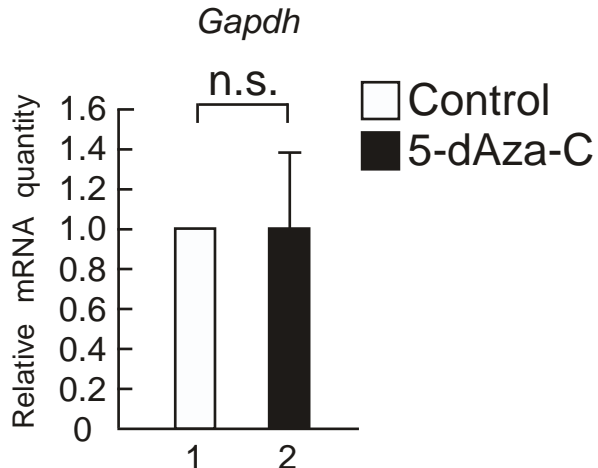

Supplement: Additional file 2 — mRNA levels of reference genes. Cyclophilin were used to normalize genes in Figure 1B and 5B. Gapdh were used to normalize genes in Figure 5C. [file 1755-8794-5-5-S2.PDF]
